# Thoracic Aorta Diameter Calculation by Artificial Intelligence Can Predict the Degree of Arterial Stiffness

**DOI:** 10.3389/fcvm.2021.737161

**Published:** 2021-12-15

**Authors:** Yaoling Wang, Jinrong Yang, Yichen Lu, Wenliang Fan, Lijuan Bai, Zhuang Nie, Ruiyun Wang, Jie Yu, Lihua Liu, Yun Liu, Linfeng He, Kai Wen, Li Chen, Fan Yang, Benling Qi

**Affiliations:** ^1^Department of Geriatrics, Union Hospital, Tongji Medical College, Huazhong University of Science and Technology, Wuhan, China; ^2^Department of Radiology, Union Hospital, Tongji Medical College, Huazhong University of Science and Technology, Wuhan, China; ^3^Siemens Healthineers Digital Technology (Shanghai) Co., Ltd., Shanghai, China; ^4^School of Software and Microelectronics, Peking University, Beijing, China; ^5^Novartis Pharmaceuticals Corporation, East Hanover, NJ, United States

**Keywords:** arterial stiffness, cardio-ankle vascular index, arterial diameter, chest computed tomography, artificial intelligence

## Abstract

**Background:** Arterial aging is characterized by decreased vascular function, caused by arterial stiffness (AS), and vascular morphological changes, caused by arterial dilatation. We analyzed the relationship of pre-AS and AS, as assessed by cardio ankle vascular index (CAVI), with arterial diameters (AD) at nine levels, from the aortic sinus to the abdominal aorta, as measured by artificial intelligence (AI) on non-enhanced chest computed tomography (CT) images.

**Methods:** Overall, 801 patients who underwent both chest CT scan and arterial elasticity test were enrolled. Nine horizontal diameters of the thoracic aorta (from the aortic sinuses of Valsalva to the abdominal aorta at the celiac axis origin) were measured by AI using CT. Patients were divided into non-AS (mean value of the left and right CAVIs [M.CAVI] < 8), pre-AS (8 ≤ M.CAVI < 9), and AS (M.CAVI ≥ 9) groups. We compared AD differences among groups, analyzed the correlation of age, ADs, and M.CAVI or the mean pressure-independent CAVI (M.CAVI_0_), Furthermore, we evaluated the risk predictors and the diagnostic value of the nine ADs for pre-AS and AS.

**Results:** The AD at mid descending aorta (MD) correlated strongest with CAVI (*r* = 0.46, *p* < 0.001) or M.CAVI_0_ (*r* = 0.42, *p* < 0.001). M.CAVI was most affected by the MD AD and by age. An increase in the MD AD independently predicted the occurrence of pre-AS or AS. For MD AD, every 4.37 mm increase caused a 14% increase in the pre-AS and AS risk and a 13% increase in the AS risk. With a cut-off value of 26.95 mm for the MD AD, the area under the curve (AUC) for identifying the risk of AS was 0.743. With a cut-off value of 25.15 mm, the AUC for identifying the risk of the stage after the prophase of AS is 0.739.

**Conclusions:** Aging is associated with an increase in AD and a decrease in arterial elasticity. An increase in AD, particularly at the MD level is an independent predictor of AS development.

## Introduction

Vascular aging is a characteristic feature of body frailty and is the pathological basis of chronic diseases of various vital organs, such as the cardiac, brain, and kidney. Arterial stiffness (AS), with the resultant structural and functional changes, is a consequence of vascular aging (1). Pathological changes related to AS occur in the vascular wall. Specifically, progressive endomyocardial thickening caused by enhanced elastin degradation and collagen deposition in the vascular medium, as well as perivascular fibrosis and abnormal extracellular matrix eventually lead to an increase in vessel diameter (2, 3). Biological changes in vessel walls during vessel diameter increase also lead to decreased vascular compliance. In previous studies, arterial dilation has not only been regarded as an independent risk factor for adverse vascular events (aneurysm and arterial dissection) but has also been considered as an independent predictor of adverse cardiovascular events (4). Adverse outcomes were strongly associated with increased AS.

The cardio ankle vascular index (CAVI) was introduced in 2006 as a method for evaluating arterial stiffness directly (5). It yielded reproducible results regardless of blood pressure (6, 7). It is derived from the Bramwell-Hill equation and introduces the stiffness parameter β. This parameter β represents arterial dilatability and correlates with changes in arterial diameter (AD) during systole and diastole (8). However, the study of Spronck et al. reported that the CAVI was not independent of blood pressure and proposed a corrected form that was independent of blood pressure, the CAVI_0_ (9, 10). They are calculated using the following equations:


$$\begin{array}{l} \beta =\frac{\ln\left(\frac{Ps}{Pd}\right)}{\frac{\Delta D}{D}};CAVI=a\times \left(2\rho \times \frac{\ln\left(\frac{Ps}{Pd}\right)}{\Delta P}\times haPWV^{2}\right)+b\\ CAVI_{0}=\frac{PWV^{2}\times 2\rho }{P_{d}}-\ln\frac{P_{d}}{P_{ref}} \end{array}$$

The study of Horinaka et al. (12) calculated the stiffness parameter β and pulse wave velocity (PWV) of the thoracic aorta by ECG-gated multi-detector row CT, and calculated the CAVI according to the formula. Moreover, the calculated CAVI was related to the CAVI determined using dynamic elasticity-measuring instruments. From the equation, it can be deduced that the parameter β is related to the AD, while CAVI or CAVI_0_ appears to be independent of the AD. Therefore, it is not clear whether arterial elasticity evaluated by CAVI is related to AD in a clinical setting. In addition, the value of CAVI is unique, based on one measurement, whereas the value of the AD varies at different levels of the artery.

Based on the above association between AD and AS, we hypothesized that morphological measurements of AD might also be effective in reflecting arterial elasticity. We measured the diameters of the thoracic aorta at nine consecutive planes in the mediastinal window of non-enhanced chest CT using artificial intelligence (AI). We also measured the CAVI at the same time. By analyzing the association between AD and pre-AS or AS, defined based on the CAVI, we assessed the effect of different levels of ADs on predicting risk of pre-AS or AS, and calculate the cut-off value of different levels of AD for identifying pre-AS or AS.

## Materials and Methods

The study protocol was reviewed and approved by Union Hospital Medical Ethics Committee. All materials were made publicly available at the HARVARD Dataverse and can be accessed at https://doi.org/10.7910/DVN/C0YY9I.

### Subjects

A total of 801 patients admitted to geriatric and general practice departments of our institution between March 2018 and October 2019 who underwent both non-enhanced chest CT and arterial elasticity measurement were enrolled. According to the 2010 clinical guideline by the American Heart Association (9), samples with significant arterial abnormalities or diseases that severely affected the structure of the arteries were excluded. Exclusion criteria are as follows:(1) a history of aortic revascularization, replacement, or stent implantation; (2) identified or suspected genetic syndromes associated with thoracic aortic aneurysms and dissections (e.g., Marfan syndrome; Turner syndrome); (3) congenital variation in an entire or important branch of the aorta in adults (e.g., abnormal right subclavian artery, aortic coarctation); (4) inflammatory diseases associated with the thoracic aortic disease; (5) acute arterial syndrome, including aortic dissection and intramural hematoma; (6) aortic Aneurysms; (7) severe insufficiency of blood volume and unstable hemodynamics; (8) severe heart failure with low ejection fraction; (9) patients on hemodialysis.

Data on age, sex, body mass index (BMI), blood pressure at admission, serum liver enzymes, kidney function, serum electrolytes, serum lipid profile, clinical diagnosis, personal history of smoking, and alcohol consumption were also collected at the same time.

### Measurement of CAVI and Calculation of CAVI_0_

A single physician measured CAVI using a VS-1000 arteriosclerosis detector (Fukuda Denshi, Tokyo, Japan). Before the examination, the patient rested quietly for approximately 15 min. CAVI was measured in a supine position. The device recorded blood pressure at both the brachia and ankles, as well as electrocardiogram, phonocardiograms, and pulse waves, and automatically calculated the CAVI on the left and right sides. Finally, the mean value of CAVI (M.CAVI) on both sides was taken for analysis.

CAVI_0_ was calculated as a corrected form of CAVI, independent of blood pressure (10, 11). We included the mean value of bilateral CAVI_0_ (M.CAVI_0_) to explore its association with ADs and age.

### Groups

Based on the recommendations of the Committee on Physiological Diagnostic Criteria for Vascular Failure (13), we divided the subjects into three groups: non-AS (M.CAVI <8), pre-AS (8 ≤ M.CAVI <9), and AS (M.CAVI ≥ 9).

Patients were divided into four age groups according to the quartile of age: age <49 years (25%), 49 years (25%) ≤ age <55 years (50%), 55 years (50%) ≤ age <66 years (75%), and age ≥ 66 years (75%).

### CT Image Acquisition and AI Post-Processing

The CT images of the entire patient cohort (*n* = 801) were captured using three different scanners (Somatom Force, Siemens, Munich, Germany; Aquilion ONE, Toshiba, Minato, Japan; CT750 HD, GE Healthcare, Chicago, IL, USA). Scans were performed from the level of the upper thoracic inlet to the inferior level of the costophrenic angle. The field-of-view depended on the size of the reconstruction frame, which varied across patients. Typically, the size of the matrix was 512 × 512 pixels. The following parameters were used: detector collimation widths 64 × 0.6 mm, or 128 × 0.6 mm, tube voltage 120 kV, and the tube current regulated by an automatic exposure control system. Therefore, when FOV was 200, the matrix was 512 × 512, the detector was 128 × 0.6, the voxel size was 0.4 × 0.4 × 0.6, and images were reconstructed with a slice thickness of 0.625–1.5 mm.

All studies were automatically processed by the research prototype AI-Rad Companion (Chest CT) offered by Siemens-Healthineers, Munich, Germany. The prototype detects the landmark of the aorta using a deep reinforcement learning algorithm dedicated to organ segmentation (14, 15). For a specific CT data set, the landmarks of the centerline of the aorta were detected, and the positions of nine specific aorta segments defined by the 2010 Clinical Guideline of the AHA were located (9). The centerline combined with the aortic landmarks was used to identify the measurement planes. For each location, the mean diameter, defined as the square root of the maximum and minimum diameters of the 2D segment, was automatically calculated. For the 801 patients, 801 CT image sets were processed, and the measurements were tabulated.

**Figure 1:**
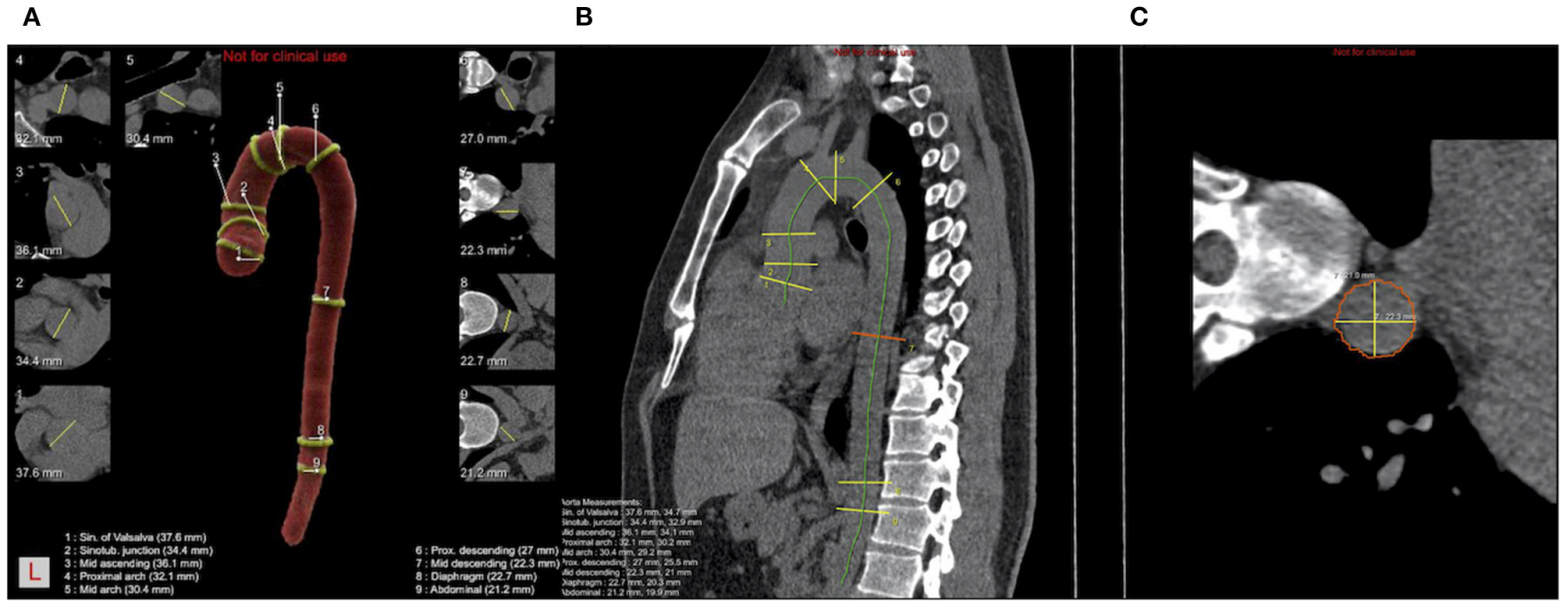
Schematic diagram of ADs calculated by AI and nine consecutive levels of positions measured. **(A)** Pseudocolor image of a thoracic aorta segmentation in a case with nine levels positions and nine levels cross-sections with yellow lines representing the longest diameter; **(B)** Schematic diagram of the sagittal plane of thoracic aorta, the green line is the centerline, yellow line and red line are the positions of nine levels; **(C)** The cross-section of MD is shown in **(B)**, where the yellow line represents the longest and shortest diameters, and the final diameter is the square root of the product of the long diameter and the short diameter.

The key landmarks and mark positions are shown below (Figure 1): (1) aortic sinuses of valsalva (SV); (2) sinotubular junction (STJ); (3) mid ascending aorta (Mas) (midpoint in length between 2 and 4); (4) proximal aortic arch (Par) (aorta at the origin of the innominate artery); (5) mid aortic arch (Mar) (between left common carotid and subclavian arteries); (6) proximal descending thoracic aorta (PD) (begins at the isthmus, ~2 cm distal to the left subclavian artery); (7) mid descending aorta (MD) (midpoint in length between 6 and 8); (8) aorta at the diaphragm (DP) (2 cm above the celiac axis origin); (9) abdominal aorta at the celiac axis origin (Ab).

### Statistical Methods

The Shapiro-Wilks was used to test whether the continuous variables followed normally distributed or not. Non-normally distributed data were presented as median with IQs, while normally distributed data were presented as mean ± SD, and classified data were presented as amounts with percentage. Compare the differences of continuous variables among the three groups by Kruskal-Wallis test (for non-normal distribution) and ANOVA test (for normal distribution), and compare the differences between any two groups by Tukey. Compare differences of classification data among groups by Chi-square test. Perform the correlation analysis between AD and variables related to AD (such as M.CAVI, age, blood pressure, and BMI), and calculate the Spearman correlation coefficient, and draw the correlation matrix. The two-dimensional scatterplots were constructed and fitted with locally weighted regression scatter smoothing method (LOESS) and linear regression to describe the relationship among AD and age as well as M.CAVI. Moreover, we calculated the correlation coefficients of linear regression equations. Estimate the odds ratios (ORs) and 95% CI of AD at each level for risk of pre-AS and AS by Logistic regression and adjust the confounding factors. Finally, receiver operating characteristics (ROC) curves were generated to evaluate the diagnostic effect of each AD to identify patients with pre-AS or AS. *P* < 0.05 was considered statistically significant. All statistics were done using R (version 3.6.3-Mac OS X 10.11, https://www.r-project.org/).

## Results

### The Distribution Differences of Clinical Baseline Data and ADs Among the AS, Pre-AS, and Non-AS Groups

Baseline data showed that age, age groups, and systolic blood pressure (SBP) were significant differences between two groups among the AS, pre-AS, and non-AS groups, while diastolic blood pressure (DBP), only differed between the non-AS and pre-AS groups (*P* = 0.012). In addition, age and SBP increased successively in the non-AS, pre-AS, and AS groups, while BMI distribution showed the opposite trend. Among the accompanying diseases, the prevalence of diabetes (DM) and coronary heart disease (CHD) showed significantly different distributions among the AS, pre-AS, and non-AS groups. The prevalence of DM in the AS group was significantly higher than that in the pre-AS and non-AS groups (Table 1).

**Table 1:** The distribution of clinical baseline data and differences among the group of AS, pre-AS, and non-AS.

	**AS**	**Pre-AS**	**Non-AS**	***p* ^*a*^**	***p* ^*b*^**	***p* ^*c*^**	***p* ^*d*^**
*N*	202	205	394				
Sex = men (%)	152 (75.2)	141 (68.8)	266 (67.5)	0.141	0.189	0.269	0.823
Age	71 [62; 82]	58 [53; 66]	50 [45; 55]	**<0.001**	**<0.001**	**<0.001**	**<0.001**
Age group (%)				**<0.001**	**<0.001**	**<0.001**	**<0.001**
<49	6 (2.97)	21 (10.2)	169 (42.9)				
49~55	13 (6.44)	53 (25.9)	111 (28.2)				
55~66	50 (24.8)	78 (38.0)	88 (22.3)				
≥66	133 (65.8)	53 (25.9)	26 (6.60)				
BMI (kg/m^2^)	23.6 (3.18)	24.8 (3.09)	24.9 (3.38)	**<0.001**	**<0.001**	**0.002**	0.873
SBP (mmHg)	159 [143; 172]	150 [136; 165]	140 [129; 153]	**<0.001**	**<0.001**	**0.001**	**<0.001**
DBP (mmHg)	79 [72; 86]	81 [73; 87]	77 [71; 85]	**0.013**	0.157	0.303	**0.012**
M.CAVI	10.05 (1.08)	8.46 (0.29)	7.09 (0.71)	**<0.001**	**<0.001**	**<0.001**	**<0.001**
M.CAVI_0_	17.89 [16.00; 20.39]	13.56 [12.76; 14.53]	10.88 [9.88; 11.57]	**<0.001**	**<0.001**	**<0.001**	**<0.001**
ALT (U/L)	20.0 [15.0; 29.0]	20.0 [16.0; 29.0]	23.0 [15.0; 34.0]	0.138	0.180	0.538	0.325
AST (U/L)	22.0 [18.0; 28.0]	21.0 [18.0; 25.0]	21.0 [18.0; 25.0]	0.156	0.157	0.157	0.941
CREA (umol/L)	78.6 [66.3; 94.4]	73.0 [64.0; 81.4]	70.4 [59.7; 79.0]	**<0.001**	**<0.001**	**<0.001**	**0.023**
Ca (mmol/L)	2.23 [2.14; 2.31]	2.22 [2.15; 2.31]	2.22 [2.16; 2.31]	0.760	0.879	0.905	0.879
P (mmol/L)	0.99 [0.89; 1.10]	1.00 [0.91; 1.13]	1.02 [0.94; 1.12]	0.098	0.097	0.292	0.475
CHOL (mmol/L)	3.96 [3.27; 4.71]	4.34 [3.66; 5.33]	4.58 [3.88; 5.19]	**<0.001**	**<0.001**	**<0.001**	0.183
TG (mmol/L)	1.15 [0.87; 1.89]	1.46 [0.99; 1.99]	1.46 [1.00; 2.38]	**0.003**	**0.002**	**0.021**	0.506
Smoke = Yes (%)	73 (36.1)	68 (33.2)	124 (31.5)	0.518	0.741	0.741	0.741
Alcohol = Yes (%)	50 (24.8)	57 (27.8)	120 (30.5)	0.336	0.518	0.562	0.562
DM = Yes (%)	75 (37.1)	43 (21.0)	59 (15.0)	**<0.001**	**<0.001**	**0.002**	**0.001**
CAD = Yes (%)	79 (39.1)	42 (20.5)	34 (8.63)	**<0.001**	**<0.001**	**<0.001**	**<0.001**
OP = Yes (%)	48 (23.8)	42 (20.5)	45 (11.4)	**<0.001**	**<0.001**	0.499	**0.006**
CLD^e^ = Yes (%)	18 (8.91)	6 (2.93)	5 (1.27)	**<0.001**	**<0.001**	**0.028**	0.199
**AD (mm)**							
SV	35.9 (4.12)	35.6 (4.30)	34.7 (4.23)	**0.001**	**0.002**	0.636	**0.042**
STJ	33.8 (3.43)	33.4 (3.92)	32.4 (4.31)	**<0.001**	**<0.001**	0.576	**0.012**
Mas	39.4 (4.27)	38.1 (4.38)	35.4 (4.50)	**<0.001**	**<0.001**	**0.008**	**<0.001**
Par	35.1 (3.43)	33.8 (3.16)	31.8 (3.59)	**<0.001**	**<0.001**	**0.001**	**<0.001**
Mar	31.7 (2.82)	31.0 (2.65)	29.2 (3.04)	**<0.001**	**<0.001**	**0.023**	**<0.001**
PD	30.2 (2.99)	29.3 (3.05)	27.4 (3.09)	**<0.001**	**<0.001**	**0.005**	**<0.001**
MD	27.6 (2.84)	26.2 (2.68)	24.3 (2.99)	**<0.001**	**<0.001**	**<0.001**	**<0.001**
DP	26.5 (2.35)	25.7 (2.28)	24.4 (2.56)	**<0.001**	**<0.001**	**0.007**	**<0.001**
Ab	25.1 (2.36)	24.2 (2.35)	23.0 (2.45)	**<0.001**	**<0.001**	**<0.001**	**<0.001**

*The differences among three groups of continuous variables were performed by ANOVA test for data with normal distribution and Kruskal-Wallis test for data with non-normal distribution, respectively. And carry out the Dunn or Tukey post-hoc comparisons between two groups for nonparametric or parametric data when there was a significant difference in the global comparison. Compare differences of classification data among groups by Chi-square test*.

a *The global comparison of variables among groups of Non-AS, Pre-AS, and AS*;

b *Post-hoc comparison between Non-AS and AS*;

c *Post-hoc comparison between Pre-AS and AS*;

d *Post-hoc comparison between Non-AS and Pre-AS*;

e *CLD (chronic lung diseases) includes asthma, cystic fibrosis, chronic bronchitis, emphysema bullae, atelectasis, and chronic obstructive pulmonary disease, etc*.

*BMI, body mass index; SBP, systolic blood pressure; DBP, diastolic blood pressure; M.CAVI, the average value of cardio ankle vascular index (CAVI) on the left and right sides; M.CAVI_0_, the average value of CAVI_0_ on left and right sides; DM, diabetes mellitus; CAD, coronary artery disease; OP, osteoporosis; CLD, chronic lung diseases; AD, artery diameter; ALT, alanine aminotransferase; AST, aspartate aminotransferase; CREA, creatinine; Ca, serum calcium; P, serum phosphorus; CHOL, total cholesterol; TG, triglycerides; SV, Aortic sinuses of Valsalva; STJ, Sinotubular junction; Mas, Mid ascending aorta (midpoint in length between STJ and Par); Par, Proximal aortic arch (aorta at the origin of the innominate artery); Mar, Mid aortic arch (between left common carotid and subclavian arteries); PD, Proximal descending thoracic aorta (begins at the isthmus, ~2 cm distal to the left subclavian artery); MD, Mid descending aorta (midpoint in length between PD and DP); DP, aorta at the diaphragm (2 cm above the celiac axis origin); Ab, Abdominal aorta at the celiac axis origin. Values with statistical differences (P <0.05) are shown in bold*.

**Figure 2:**
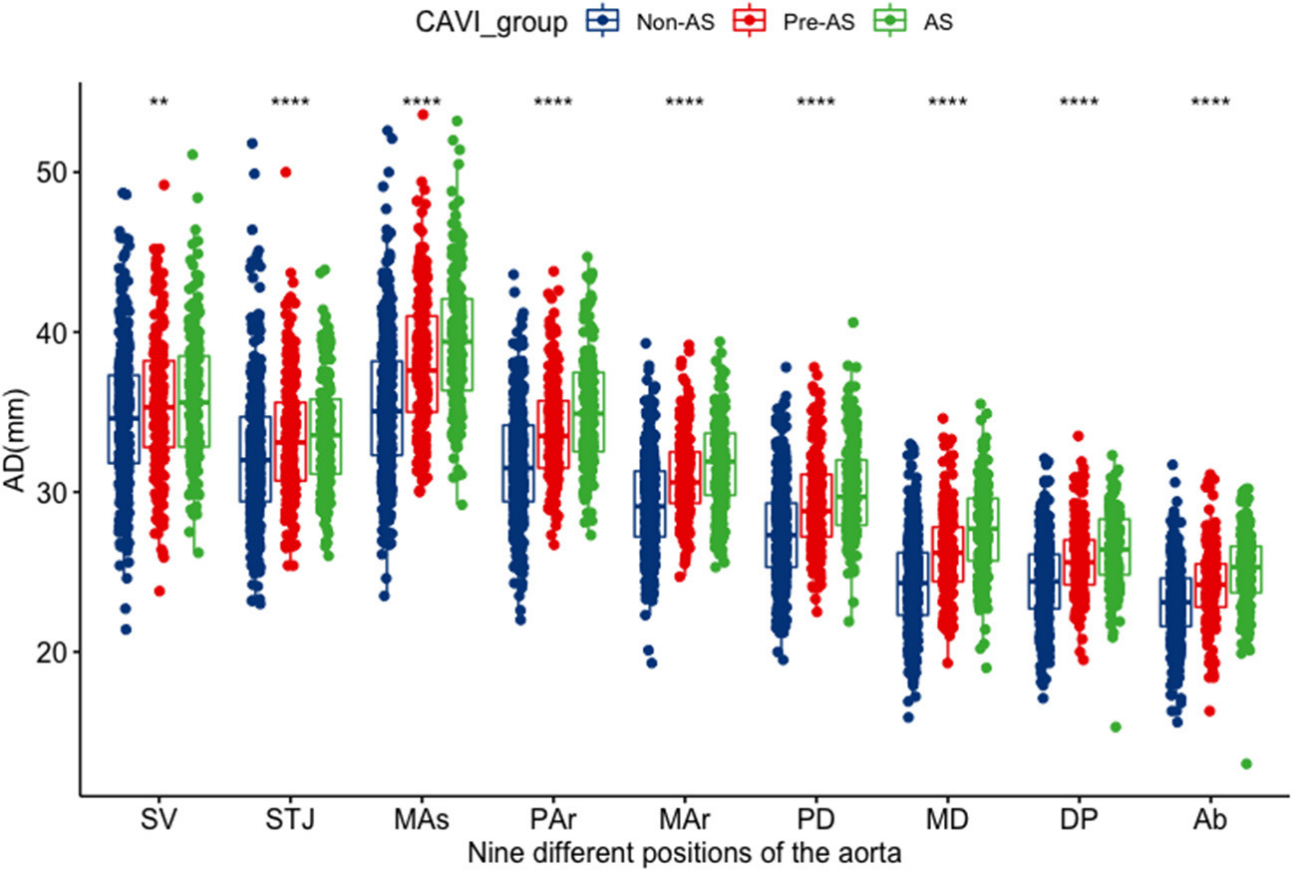
The distribution differences of the AD at various levels among the groups of non-AS, pre-AS, and AS. SV, aortic sinuses of valsalva; STJ, sinotubular junction; Mas mid ascending aorta (midpoint in length between STJ and Par); par, proximal aortic arch (aorta at the origin of the innominate artery); Mar, mid aortic arch (between left common carotid and subclavian arteries); PD, proximal descending thoracic aorta (begins at the isthmus, ~2 cm distal to left subclavian artery); MD, mid descending aorta (midpoint in length between PD and DP); DP, aorta at the diaphragm (2 cm above the celiac axis origin); Ab, abdominal aorta at the celiac axis origin. *It represents there was a statistical difference between any two of the three groups; **P < 0.01; ****P < 0.001.

There were no differences in ADs at the sinus of valsalva and the sinotubular junction between AS and pre-AS groups. Other levels of ADs showed significant differences among the non-AS, pre-AS, and AS groups by pair-wise comparison: ADs increased in the order of the non-AS, pre-AS, and AS groups (Table 1). The ADs narrowed from the SV to the STJ, then widened, and reached a maximum at the MAs. Subsequently, it decreased again (Figure 2).

### Correlation of ADs With M.CAVI, M.CAVI_0_, Age, BMI, and Blood Pressure

#### Correlation Matrix for ADs, M.CAVI, M.CAVI_0_, Age, BMI, and Blood Pressure

The ADs at all levels were positively correlated with age, BMI, M.CAVI, M.CAVI_0_, and blood pressure (BP; both SBP and DBP). There was a weak correlation between the ADs at the SV and STJ and M. CAVI (*r* = 0.14–0.16) or M.CAVI_0_ (*r* = 0.1–0.12), but the strong correlation between the AD at subsequent levels (from MAs to the abdominal aorta at the celiac axis origin[Ab]) and M. CAVI (*r* = 0.36–0.46) or M.CAVI_0_ (*r* = 0.32–0.42). The correlation between the AD at the mid-descending aorta (MD) and M.CAVI or M.CAVI_0_ was is the strongest (*R* = 0.46 and 0.42, respectively). There was also a significant correlation between any two levels of AD (*r* = 0.5–0.91). The Spearman correlation coefficient of M. CAVI and age was 0.64 (Supplementary Figure 1).

#### Association Between M.CAVI and ADs Adjusted by Age_Group

**Figure 3:**
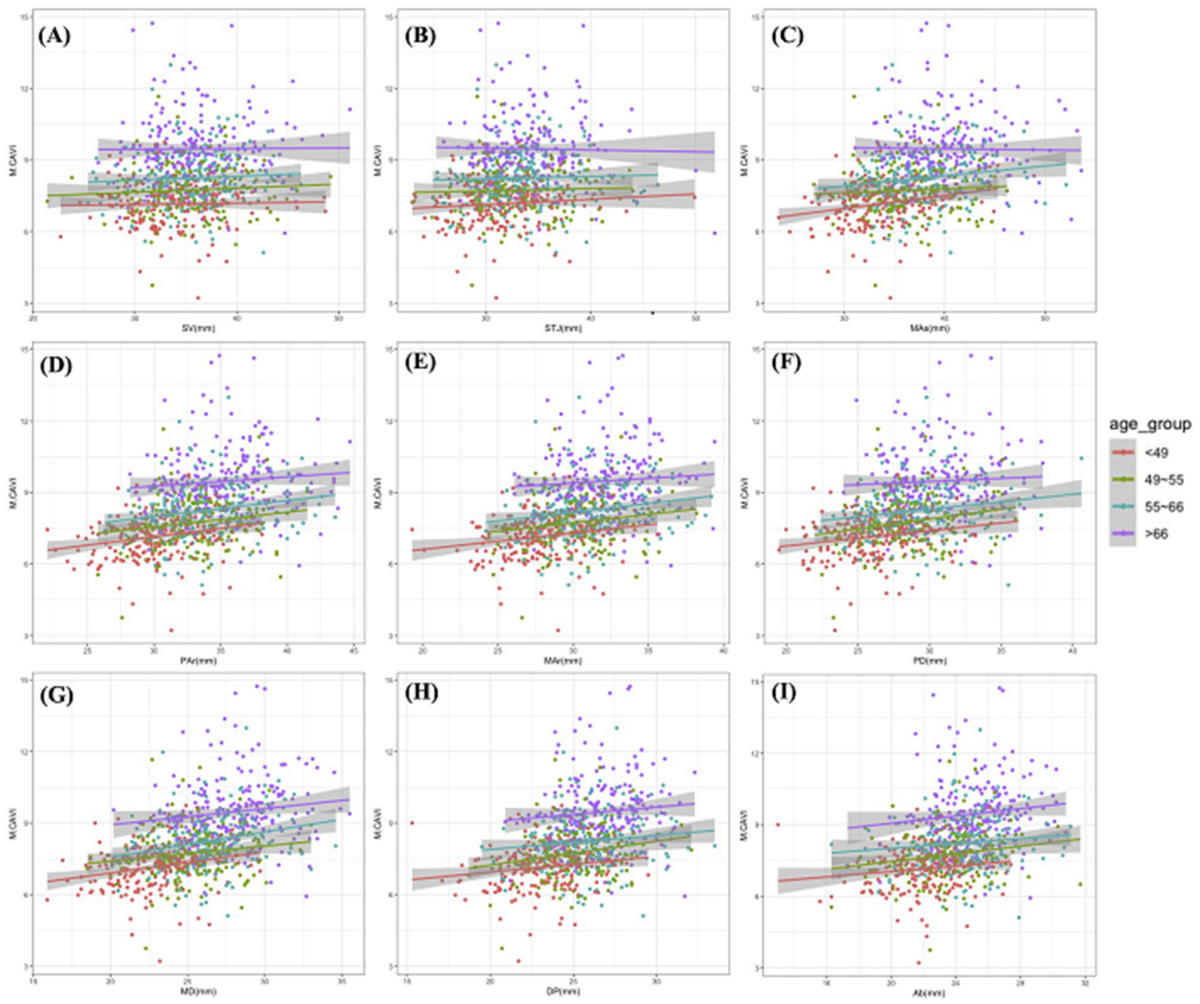
The scatter plots and simple linear fitting curves of M.CAVI with ADs by different age groups. All scatter plots were drawn with M.CAVI as the y-axis, and AD at SV **(A)**, STJ **(B)**, Mas **(C)**, Par **(D)**, Mar **(E)**, PD **(F)**, MD **(G)**, DP **(H)**, Ab **(I)** as the x-axis separately. Abbreviations are the same as Figure 2.

M.CAVI increased with an increase in the ADs at different levels. Older age groups had a generally higher M.CAVI than the younger age groups (Figure 3). The increase in M. CAVI was more rapid with the increase in ADs (with fitting functions having a larger slope) in the younger than in the older age groups. According to the regression coefficient fitted by multivariate linear regression, M.CAVI was most affected by the AD of the MD, even after adjusted for age_group (Table 2).

**Table 2:** Multivariate linear regression between M.CAVI/M.CAVI_0_ and AD at 9 positions.

		**Regression coefficients (95% CI)^a^**	**Adjust *R-*squared^a^**	***p* ^*a*^**	**Regression coefficients (95% CI)^b^**	**Adjust *R*-squared^b^**	***P* ^b^**
Model 1	SV	0.010 (−0.009~0.029)	0.36	0.303	−0.009 (−0.062~0.044)	0.40	0.750
	STJ	0.008 (−0.012~0.028)	0.36	0.430	−0.021 (−0.077~0.035)	0.40	0.466
	Mas	0.025 (0.005~0.045)	0.36	**0.012**	0.028 (−0.026~0.083)	0.40	0.305
	Par	0.058 (0.034~0.083)	0.38	**<0.001**	0.105 (0.037~0.173)	0.41	**0.003**
	Mar	0.062 (0.033~0.091)	0.37	**<0.001**	**0.106 (0.025** **~** **0.187)**	**0.41**	**0.011**
	PD	0.057 (0.030~0.084)	0.37	**<0.001**	**0.099 (0.024** **~** **0.175)**	**0.41**	**0.010**
	MD	0.081 (0.051~0.111)	0.38	**<0.001**	**0.149 (0.064** **~** **0.233)**	**0.41**	**<0.001**
	DP	0.070 (0.035~0.105)	0.37	**<0.001**	**0.133 (0.035** **~** **0.230)**	**0.41**	**0.008**
	Ab	0.066 (0.031~0.101)	0.37	**<0.001**	**0.137 (0.039** **~** **0.234)**	**0.41**	**0.006**
Model 2	SV	−0.005 (−0.025~0.015)	0.43	0.623	−0.020 (−0.074~0.034)	0.51	0.465
	STJ	−0.002 (−0.023~0.019)	0.43	0.867	−0.011 (−0.067~0.046)	0.51	0.716
	Mas	0.002 (−0.004~0.036)	0.43	0.118	0.028 (−0.026~0.081)	0.51	0.313
	Par	0.044 (0.018~0.070)	0.44	**0.001**	0.098 (0.029~0.167)	0.51	**0.005**
	Mar	0.050 (0.020~0.080)	0.44	**0.001**	0.113 (0.032~0.194)	0.51	**0.006**
	PD	0.048 (0.019~0.076)	0.44	**<0.001**	0.108 (0.031~0.186)	0.51	**0.006**
	MD	0.073 (0.038~0.108)	0.44	**<0.001**	**0.172 (0.078** **~** **0.266)**	**0.51**	**<0.001**
	DP	0.055 (0.018~0.093)	0.44	**0.004**	0.117 (0.017~0.217)	0.51	**0.022**
	Ab	0.050 (0.013~0.086)	0.44	**0.007**	0.109 (0.011~0.207)	0.51	**0.030**

a *Multivariable linear regression model with M.CAVI as the dependent variable*.

b *Multivariable linear regression model with M.CAVI_0_ as the dependent variable*.

*Model 1. Multivariable linear regression model with ADs and age_group as independent variables*.

*Model 2. Multivariate linear regression model with ADs and age_group as independent variables and adjusted by sex, BMI, SBP, DBP, CREA, CHOL, TG, DM, CAD, OP, CLD. Abbreviations are the same as the notes in Table 1. Values with statistical differences (P <0.05) are shown in bold*.

#### Association Between M.CAVI/M.CAVI_0_ and Age

In the scatter plot fitted by locally weighted regression scatter smoothing method with M.CAVI as the independent variable and age as the dependent variable, the M.CAVI increased smoothly with age (Supplementary Figure 2A). Supplementary Figure 2B shows that the scatter gram fitted by linear regression (Table 3). A similar trend was found between M.CAVI_0_ and age (Supplementary Figures 2C,D and Table 3).

**Table 3:** Simple linear regression between the AD and age or between M.CAVI and age.

	**Regression coefficients**	**95% CI**	**Adjust *R*-squared**	***P***
SV^a^	0.067	0.047~0.088	0.049	<0.001
STJ^a^	0.080	0.061~0.099	0.077	<0.001
Mas^a^	0.187	0.168~0.206	0.311	<0.001
Par^a^	0.138	0.122~0.153	0.247	<0.001
Mar^a^	0.109	0.096~0.122	0.248	<0.001
PD^a^	0.113	0.099~0.127	0.235	<0.001
MD^a^	0.136	0.123~0.148	0.360	<0.001
DP^a^	0.091	0.080~0.102	0.246	<0.001
Ab^a^	0.087	0.076~0.098	0.230	<0.001
M.CAVI^b^	0.064	0.059~0.070	0.040	<0.001
M.CAV${\text{I}}_{0}^{\text{c}}$	0.195	0.180~0.210	0.450	<0.001

a *Simple linear regression with age as the independent variable and the AD at 9 levels as the dependent variable*;

b *Simple linear regression with age as the independent variable and M.CAVI as the dependent variable*;

c *Simple linear regression with age as the independent variable and M.CAVI_0_ as the dependent variable. The abbreviations are the same as the notes in Table 1*.

#### Association Between ADs and Age

The ADs at all levels expanded with age, although at different rates. As shown in Supplementary Figure 3, the AD at the MD increased most rapidly with age, While the AD at the SV increased the slowest with age (Table 3, regression coefficients = 0.136 and 0.067, respectively).

#### Association of M.CAVI/M.CAVI_0_, With the AD at the MD, and Age

A three-dimensional scatter plot displays the interaction between age, M.CAVI/M.CAVI_0_, and the AD at the MD (Supplementary Figures 4A,B). The planes were fitted by a regression equation (M.CAVI-MD + age and M.CAVI_0_-MD + age), respectively. As the fitted plane rises from the lower left to the upper right on the plot, the darker the scatter, the larger the value, which indicates that, with the increase in age, M.CAVI/M.CAVI_0_ increased, and the AD at the MD also increased.

### Logistic Regression Analysis of AD at Each Level to Predict the Risk of AS (M.CAVI ≥ 9), or the Risk of Pre-AS and AS (M.CAVI ≥ 8)

In Model 1, an increase in ADs at all levels showed a significant risk of AS [odds ratio (OR) > 1, *P* < 0.05]. The increase in the AD at the MD had the strongest predictive effect on the risk of AS, pre-AS and AS (OR = 1.349 and 1.362, respectively). In Model 2, which was adjusted by age group, and in Model 3, which was adjusted by clinical baseline data, ADs at some levels were no longer significant in predicting the risk of AS or pre-AS and AS, and increased AD at MD remained at high risk. With every 4.37 mm increase in the AD at the MD, the risk of developing pre-AS and AS increased by 14%, and the risk of developing AS increased by 13% (Table 4).

**Table 4:** Logistic regression analysis of the AD at each level to predict the risk of AS, pre-AS and AS.

	**Model 1**			**Model 2**			**Model 3**		
	**OR**	**95% CI**	***P***	**OR**	**95% CI**	***P***	**OR**	**95% CI**	***P***
SV^a^	1.054	1.015~1.095	0.006	1.004	0.960~1.050	0.872	0.970	0.921~1.022	0.251
STJ^a^	1.065	1.024~1.107	0.002	0.996	0.948~1.045	0.857	0.976	0.921~1.033	0.408
Mas^a^	1.152	1.112~1.196	<0.001	1.022	0.977~1.069	0.336	1.004	0.954~1.056	0.889
Par^a^	1.218	1.162~1.279	<0.001	1.073	1.014~1.136	0.015	1.041	0.974~1.113	0.232
Mar^a^	1.236	1.168~1.310	<0.001	1.066	0.995~1.142	0.068	1.037	0.957~1.125	0.374
PD^a^	1.233	1.170~1.301	<0.001	1.078	1.012~1.150	0.021	1.076	0.996~1.163	0.062
MD^a^	1.349	1.270~1.438	<0.001	1.125	1.046~1.211	0.002	1.105	1.005~1.215	0.040
DP^a^	1.302	1.215~1.398	<0.001	1.067	0.981~1.161	0.130	1.024	0.925~1.133	0.645
Ab^a^	1.346	1.252~1.452	<0.001	1.111	1.022~1.210	0.015	1.086	0.985~1.200	0.099
SV^b^	1.062	1.027~1.099	<0.001	1.012	0.972~1.054	0.565	0.988	0.942~1.037	0.625
STJ^b^	1.077	1.040~1.117	<0.001	1.009	0.967~1.053	0.686	0.993	0.945~1.044	0.793
Mas^b^	1.187	1.146~1.231	<0.001	1.062	1.018~1.108	0.005	1.043	0.995~1.094	0.080
Par^b^	1.247	1.192~1.306	<0.001	1.099	1.042~1.160	<0.001	1.077	1.013~1.145	0.017
Mar^b^	1.285	1.219~1.358	<0.001	1.110	1.043~1.182	0.001	1.093	1.018~1.175	0.015
PD^b^	1.277	1.214~1.346	<0.001	1.118	1.055~1.187	<0.001	1.111	1.038~1.192	0.003
MD^b^	1.362	1.289~1.444	<0.001	1.139	1.067~1.219	<0.001	1.126	1.035~1.277	0.006
DP^b^	1.334	1.253~1.424	<0.001	1.097	1.018~1.183	0.016	1.063	0.974~1.161	0.175
Ab^b^	1.336	1.253~1.428	<0.001	1.101	1.023~1.187	0.011	1.076	0.988~1.174	0.095

*Model 1 no adjustments have been made; Model 2 adjusted by age group; Model 3 adjusted by age group, sex, BMI, SBP, DBP, CREA, CHOL, TG, DM, CAD, OP, and CLD*.

a *The changes of ADs to predict the risk of AS (i.e., M.CAVI ≥ 9)*;

b *The changes of ADs to predict the risk of pre-AS and AS (i.e., M.CAVI ≥ 8)*.

*OR, odds ratios; CI, confidence intervals; The remaining abbreviations are the same as the notes in Table 1*.

### ROC Curve to Evaluate the Efficacy of ADs in the Diagnosis of AS (M.CAVI ≥ 9) and Pre-AS and AS (M.CAVI ≥ 8)

**Figure 4:**
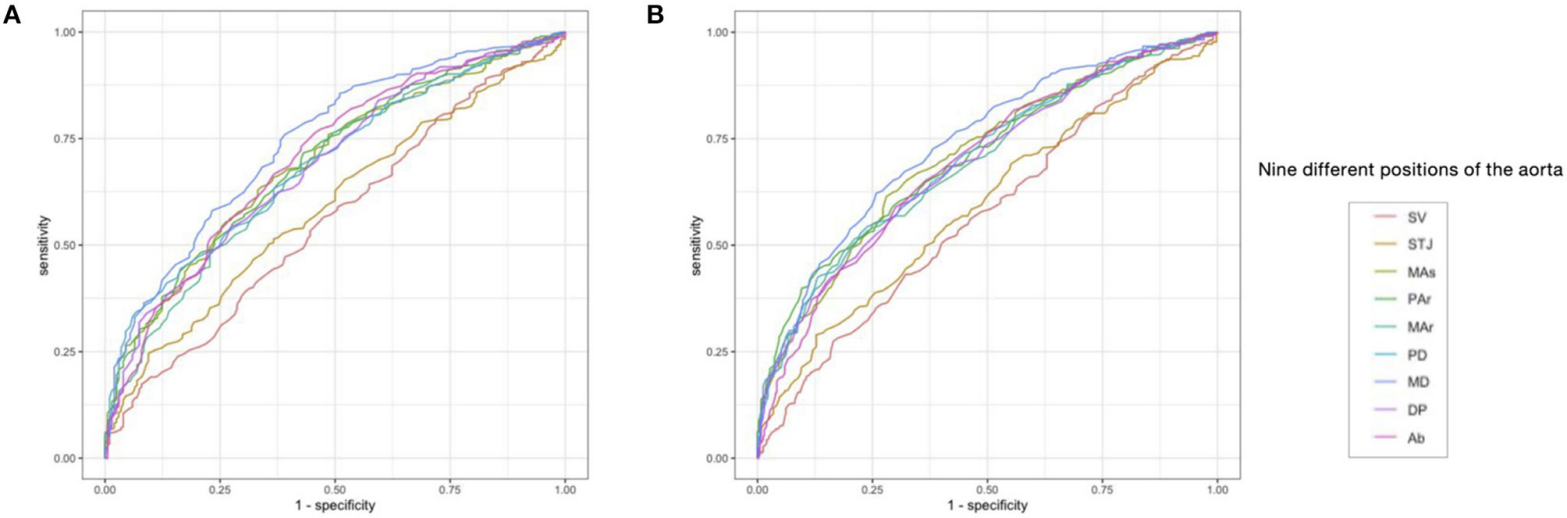
The receiver operating curve (ROC) of AD at each level for predicting the risk of AS, and the risk of Pre-AS and AS. **(A)** Identification of the risk of AS (i.e., M.CAVI ≥ 9) by the AD. **(B)** Identification of the risk of pre-AS and AS (i.e., M.CAVI ≥ 8) by the AD. Abbreviations are the same as Figure 2.

The ADs at SV and STJ were less effective in identifying AS or pre-AS and AS (AUC = 0.559–0.594), while the ADs at the other seven levels had relatively good diagnostic performance (AUC = 0.678–0.743). The AUC of the AD at the MD for identifying AS or pre-AS and AS was 0.743 (0.704–0.781) and 0.739 (0.705–0.773), respectively. The cut-off values were 26.95 and 25.15 mm, respectively. For the identification of AS, the ADs at the MAr, MD, and Ab had high specificity (76.0–76.3%), while the ADs at the SV and STJ had the best sensitivity (92.1 and 90.6%, respectively) (Figure 4 and Table 5).

**Table 5:** Evaluation of the predictive effect of the AD at each level on the risk of AS and risk of pre-AS and AS by the receiver operating characteristic curves (ROC).

	**AUC (95% CI)**	**Specificity**	**Sensitivity**	**Cut-off value**	**Youden index**
SV^a^	0.559 (0.514~0.604)	0.174	0.921	31.05	0.095
STJ^a^	0.590 (0.547~0.633)	0.245	0.906	29.75	0.151
MAs^a^	0.693 (0.653~0.733)	0.629	0.668	37.45	0.297
PAr^a^	0.697 (0.657~0.737)	0.716	0.569	34.25	0.285
MAr^a^	0.678 (0.636~0.719)	0.763	0.505	31.85	0.268
PD^a^	0.691 (0.652~0.730)	0.444	0.837	27.45	0.281
MD^a^	0.743 (0.704~0.781)	0.760	0.609	26.95	0.369
DP^a^	0.687 (0.646~0.728)	0.541	0.728	25.05	0.269
Ab^a^	0.703 (0.662~0.745)	0.763	0.545	25.05	0.308
SV^b^	0.570 (0.531~0.610)	0.431	0.678	33.75	0.109
STJ^b^	0.594 (0.555~0.633)	0.289	0.872	29.75	0.161
Mas^b^	0.708 (0.672~0.743)	0.641	0.720	36.15	0.361
PAr^b^	0.705 (0.669~0.741)	0.444	0.867	30.85	0.311
MAr^b^	0.694 (0.658~0.730)	0.515	0.789	29.15	0.304
PD^b^	0.701 (0.665~0.737)	0.543	0.762	27.55	0.305
MD^b^	0.739 (0.705~0.773)	0.622	0.742	25.15	0.364
DP^b^	0.691 (0.655~0.727)	0.594	0.688	24.95	0.282
Ab^b^	0.693 (0.657~0.729)	0.589	0.700	23.55	0.289

a *The predictive effect of ADs on AS risk (i.e., M.CAVI ≥ 9)*;

b *The predictive effect of ADs on the risk of pre-AS and AS (i.e., M.CAVI ≥ 8)*.

*AUC, the area under the curve; CI, confidence intervals; The remaining abbreviations are the same as the notes in Table 1*.

## Discussion

### Major Findings of This Study

Except for the ADs at the SV and STJ, which showed no significant difference between the AS and pre-AS groups, the ADs at other levels showed significant differences among the non-AS, pre-AS, and AS groups by pair-wise comparison. All levels of ADs increased in the non-AS, pre-AS, and AS groups, in that order. There was a weak correlation between M.CAVI and the ADs at the start of the thoracic aorta (SV, STJ), and a strong correlation between M.CAVI and ADs at subsequent levels. In particular, the AD at the MD showed the strongest correlation with M. CAVI. CAVI_0_ behaved similarly to CAVI. The older age groups had higher M.CAVI and M.CAVI_0_ than the younger age groups. The M.CAVI increased with the increase in ADs at different levels, and this increase was more rapid in younger people. An increase in AD at all levels was associated with a significant AS risk. In particular, the increase in the AD at the MD could be considered as an independent risk factor for AS, pre-AS, and AS, after adjusting for confounders. In the age-adjusted model, for each 4.37 mm increase in the AD at the level of the MD, the risk of pre-AS and AS increased by 14%, and the risk of AS increased by 13%. At the optimal cut-off value of the AD at the MD (26.95 mm), the AUC for identifying AS was 0.743. When this value was 25.15 mm, the AUC for identifying pre-AS and AS was 0.739.

### AD Increases With Aging

The increase in AD and the decline in arterial elasticity both reflect age-related degeneration of vessels in terms of morphology and function. Previous studies have determined systemic changes in ADs with age, which occur from the peripheral to central arteries (16). Changes in carotid AD have attracted much attention in the past since they could be measured effortlessly. The study of Bruneck et al. (17) found that vessels appeared dilated with age when vessel wall thickness exceeded the 50th percentile of the population. That is, once the intima-media thickness of the common carotid artery exceeded 0.75 mm, or that of the internal carotid artery exceeded 0.9 mm. A cross-sectional study of 69 men aged 16–75 years also confirmed that the common carotid AD increased almost linearly with age (*r* = 0.46, *p* < 0.001) (18). In this study, the ADs at different levels were also correlated with age (*r* = 0.22–0.6, *p* < 0.001). Another study in ARICLAD found a significant positive correlation between age and AD at the common carotid artery after adjusting for other risk factors, and this effect was more pronounced in people with manifestations of AS. The AD dilation rate increased with the increased risk of AS (0.017–0.03 mm/year) (19). In our study, the AD dilation rate of the aorta at all levels was 0.067–0.187 mm/year.

### AS Develops With Aging

The reasons for the age-related changes in AD are manifold. Elastic fibers in the arterial wall are lost with age, leaving a hard elastic layer. The elastic layer separates due to the loss of elastic fibers, and proteoglycan fills in the resulting gap, which eventually leads to the thickening of the arterial wall and dilation of the artery (increase in AD) (20). The relationship between the development of AS and aging has been studied using a variety of arterial elasticity assessment methods: Carotid-femoral PWV and brachial-ankle PWV were found to increase with age in the ARIC study of 4,974 older subjects (21). A study of older adults (over 65 years of age) found that the average carotid intima-media thickness increased by 1.53 um/year (22). Studies in healthy people have found that CAVI increases with age and has a linear regression coefficient of 0.045 in women while 0.048 in men, and that it is a sensitive marker of AS progression (23). In this study, the regression coefficient of CAVI with age was 0.064, and a rapid and stable increase in CAVI was seen with age between 37 and 75 years. This suggests that therapeutic interventions for AS maybe even more necessary during this period. The loss of elastic fibers in the arterial wall with age not only leads to an increase in AD but also leads to a decline in arterial elasticity. In addition, the accumulation of advanced glycation end products (AGEs) with aging leads to the cross-linking of collagen and elastin, resulting in a decrease in arterial wall tissue ductility (24, 25). AGE-AGE receptor interactions also impair endothelial function.

### Interaction Between AS and AD

Therefore, an increase in AD and a decrease in arterial elasticity together constitute the overall degeneration of vascular aging and are mutually enhancing. Previous studies on AD and decreased vascular function have focused on the relationship between vascular diameter and vasodilation, as well as the relationship between the wall-lumen ratio and vasodilation (26, 27). Other studies have found an association between brachial AD and carotid plaque formation (28), and a recent study has found that abdominal aortic diameter increase is a good predictor of carotid artery stenosis risk (29). However, CAVI also is considered to be associated with the risk of cardiovascular events (30–33). CAVI-related criteria for vascular failure have been proposed by the Physiological Diagnosis Criteria for Vascular Failure Committee (13). Therefore, we separated in this study the population into non-AS (M.CAVI <8), pre-AS (8 ≤ M.CAVI <9), and AS (M.CAVI ≥ 9), and further calculated the cut-off values of the ADs to predict M.CAVI ≥ 8 (pre-AS and AS) and CAVI > 9 (AS). This is of great value in exploring the clinical significance of measuring thoracic AD by AI. When the AD of the MD exceeds 25.15 mm, care is needed to decrease the risk of M.CAVI > 8 that it predicts. When the AD of the MD exceeds 26.95 mm, the risk of CAVI > 9 should be considered and addressed.

### Clinical Application of AI in Measuring AD

Using artificial intelligence algorithms based on deep reinforcement learning has been a recent trend in the analysis of 3D CT images. Trained on a multitude of datasets based on thorax scans for CT, the AI-Rad Companion research prototype (Siemens Healthineers) was able to largely automate the process of aortic measurement, a tedious yet important task more often than not neglected by radiologists. Normally, quantitative assessment of aortic measurement consists of first identifying/highlighting the contour of the aortic landmark and second locating/measuring the segments of the aortic root, aortic arch center, important bifurcations, and aorta at the diaphragm, among others. A senior radiologist in our institution with years of training in post-processing and clinical diagnosis approximately takes 7–10 min to complete such a task for one patient set. On the other hand, the algorithm was able to correctly identify the aortic landmark for both enhanced and non-enhanced scans and quickly generate visionary insights within 1–2 min for one patient (Figure 1, Example of aortic measurements for patients). With the help of AI software, batch processing of the patient cohort of 1,500 cases have now been feasible and radiologists can simply proofread the AI-generated results.

Following the AHA guideline (9), the key mark positions from root to end consist of aortic sinuses of Valsalva, sinotubular junction, proximal aortic arch, mid aortic arch, and proximal descending thoracic aorta and aorta at the diaphragm, the first 3 of which have been considered as indicators of early signs of aortic enlargement and cardiovascular risk. In our study, except that the arterial diameters of SV and STJ levels showed no difference between AS and pre-AS groups, the arterial diameters of other levels showed significant differences among the three groups of non-AS, pre-AS, and AS groups by pair comparison, and MD segment had the highest correlation with M.CAVI. Image-based deep learning algorithms are able to capture the slight fluctuation of the contour and therefore capture the clinical development of the disease progression as reflected in the aortic measurement in our case. Due to individual differences in patients, the aortic shape could vary. However, a general trend has been found taking into account several clinical segments. Being trained on the thousands of datasets of patients with various geographic backgrounds, the algorithm was able to pinpoint relevant aortic segments thereby mitigating the subjectiveness of radiologists.

### Limitations of AD Measured by AI

For AI-assisted calculation of aortic measurement, several caveats are to be heeded. The performance of the algorithm is also contingent on good scanning protocols and imaging quality. We have found in several cases where the algorithm was unable to detect the landmark due to defects in the CT images. There were also cases where the first 2 segments seemed to be incorrectly labeled due to motion and metal artifacts. Furthermore, the algorithm was trained largely on patients with normal physiology. Therefore, for patients with extreme conditions or exocentric aortic anatomy, the results should be judged with extra caution since they could be extrapolated beyond the statistical domain where outline effects could prevail. It is advised radiologists do perform a final check to confirm the result so as to avoid the potential erroneous outcomes. On the other hand, the measurement of aortic segments was simply the start of quantitative analysis of aortic conditions. Other clinical information such as volume of the aortic segment, density of aortic boundary, the thickness of the specific segments could also be crucial in inferring the cardiovascular conditions for the patient. Several of these measurements (such as volumes, densities) could be largely visible of the CT images, however, the quantification has been difficult due to a variety of imaging protocols, reconstruction kernels, clinical habits used to generate the image itself.

## Conclusion

In this study, we evaluated the relationship of ADs, measured by AI at continuous levels from the aortic sinus to the abdominal aorta, with the risk of pre-AS or AS, and quantitatively described the pairwise interaction of ADs, CAVI, and age, which is essential for a comprehensive understanding of vascular aging. Our results are consistent with previous studies showing that aging is associated with increased AD and decreased arterial elasticity. We found that increased AD, particularly in the MD, is a good predictor of AS. Thus, computer-aided calculation of AD on chest CT images may be a significant predictor of the risk of cardiovascular disease and other chronic diseases in the future, although the clinical implementation of this finding will require further verification.

## Data Availability Statement

The datasets presented in this study can be found in online repositories. The names of the repository/repositories and accession number(s) can be found at: Harvard Dataverse, https://doi.org/10.7910/DVN/C0YY9I.

## Ethics Statement

The studies involving human participants were reviewed and approved by the Union Hospital Medical Ethics Committee. Written informed consent was not required for this study, in accordance with the local legislation and institutional requirements.

## Author Contributions

JYa, YW, YLu, YLi, LL, RW, LB, WF, ZN, JYu, and LH: methodology and data collection. YW, JYa, YLu, and KW: data curation, formal analysis, visualization, and writing of the original draft. YW: supervision and writing with review and editing. LC: language polishing and statistical consultant. All authors contributed to the article and approved the submitted version.

## Funding

This study was supported by the National Natural Science Foundation of China (Grant Nos. 81571373, 81601217, and 82001491), Natural Science Foundation of Hubei Province of China (Grant No. 2017CFB627), Health Commission of Hubei Province scientific research project (WJ2021M247), and Scientific Research Fund of Wuhan Union Hospital (Grant No.2019).

## Conflict of Interest

LC was employed by company Novartis Pharmaceuticals Corporation. YL is employed by Siemens Healthineers Digital Technology (Shanghai). The remaining authors declare that the research was conducted in the absence of any commercial or financial relationships that could be construed as a potential conflict of interest.

## Publisher's Note

All claims expressed in this article are solely those of the authors and do not necessarily represent those of their affiliated organizations, or those of the publisher, the editors and the reviewers. Any product that may be evaluated in this article, or claim that may be made by its manufacturer, is not guaranteed or endorsed by the publisher.
