# Supplementary material for: Thoracic Aorta Diameter Calculation by Artificial Intelligence Can Predict the Degree of Arterial Stiffness
Source: Front Cardiovasc Med. 2021 Dec 15;8:737161. doi: 10.3389/fcvm.2021.737161 (PMC8714774; doi:10.3389/fcvm.2021.737161)
Supplement: Supplementary file 1 [file Presentation_1.PDF]

Supplementary Figure 1 The correlation matrix among the age, BMI, M.CAVI, M.CAVI$_0$, SBP, DBP, and the ADs of each level

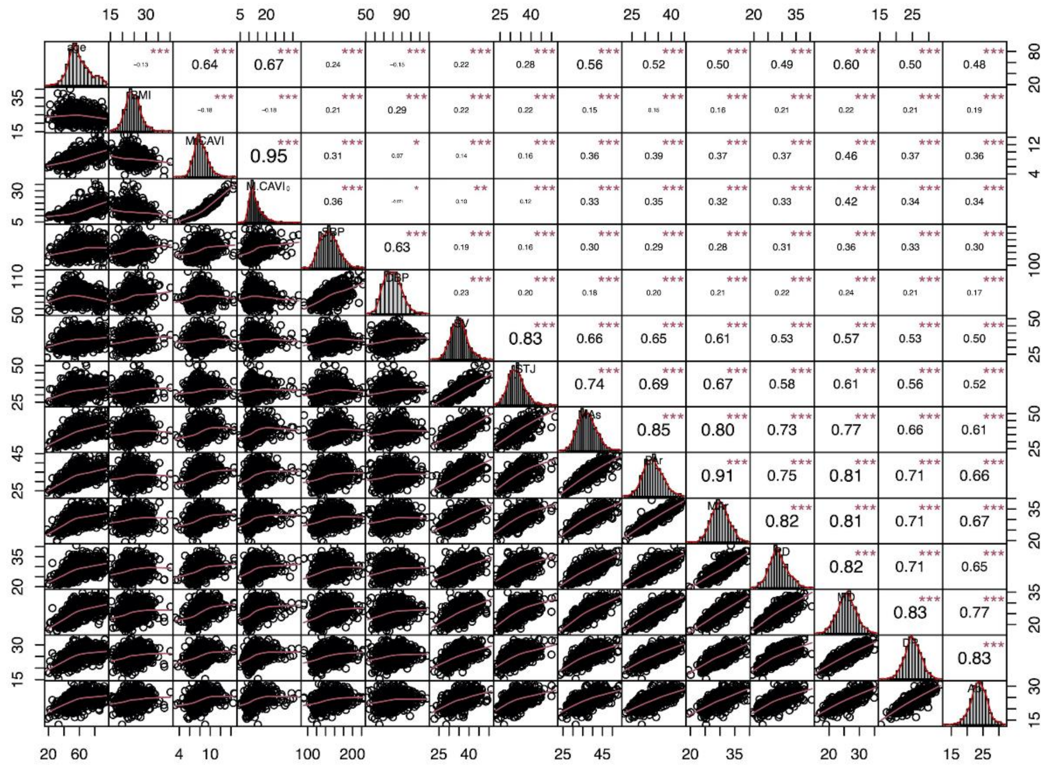

The diagonal line displays the distribution of the variables, the lower-left corner shows the bivariate scatter plot with the fitted line, and the upper right corner shows the correlation coefficient and significance level.

*, P <0.05; **, ***, P <0.01

M.CAVI The average value of the cardio ankle vascular index at the both side; SBP systolic blood pressure; DBP diastolic blood pressure; other abbreviations are the same as Fig2

Supplementary Figure 2 The scatter plot and fitting curve of M.CAVI/M.CAVI$_0$ with age

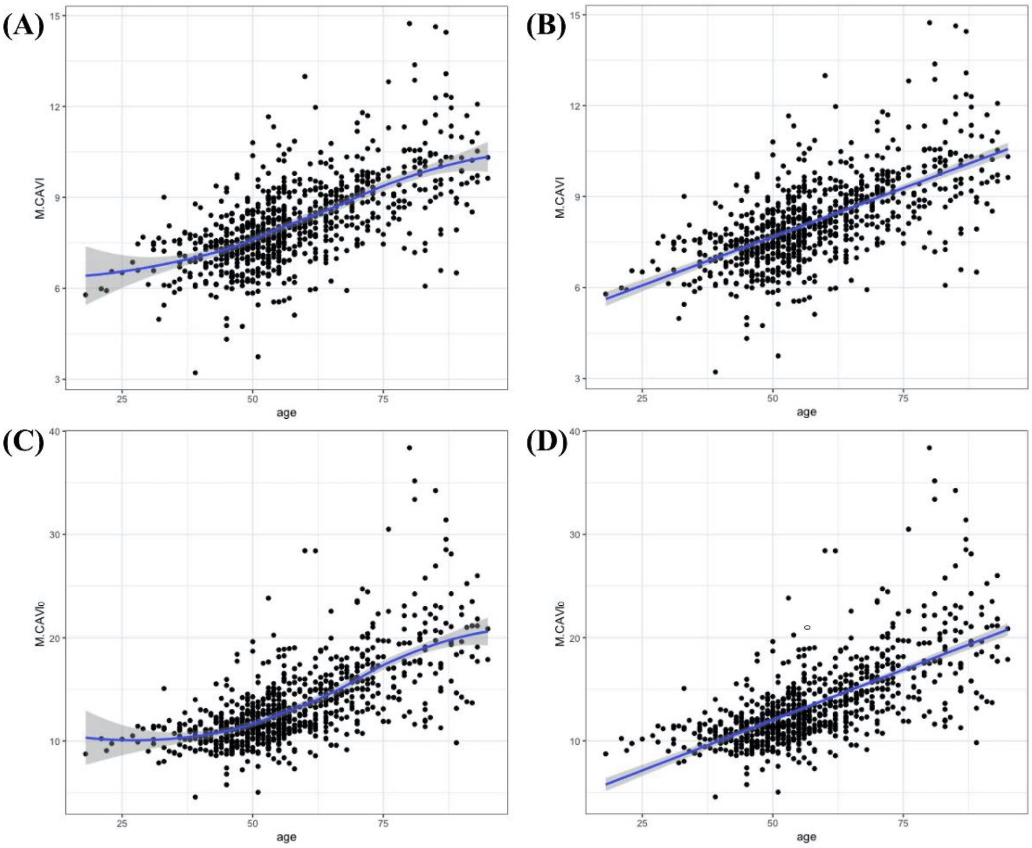

(A)The scattergram fitted by LOESS, with age as the independent variable, and M.CAVI as the dependent variable

(B) The scattergram fitted by linear regression, with age as the independent variable, and M.CAVI as the dependent variable

(C)The scattergram fitted by LOESS, with age as the independent variable, and M.CAVI$_0$ as the dependent variable

(D) The scattergram fitted by linear regression, with age as the independent variable, and M.CAVI$_0$as the dependent variable

Abbreviations are the same as Fig2

Supplementary Figure 3 The scatter plot and fitting curve of age with ADs at various positions

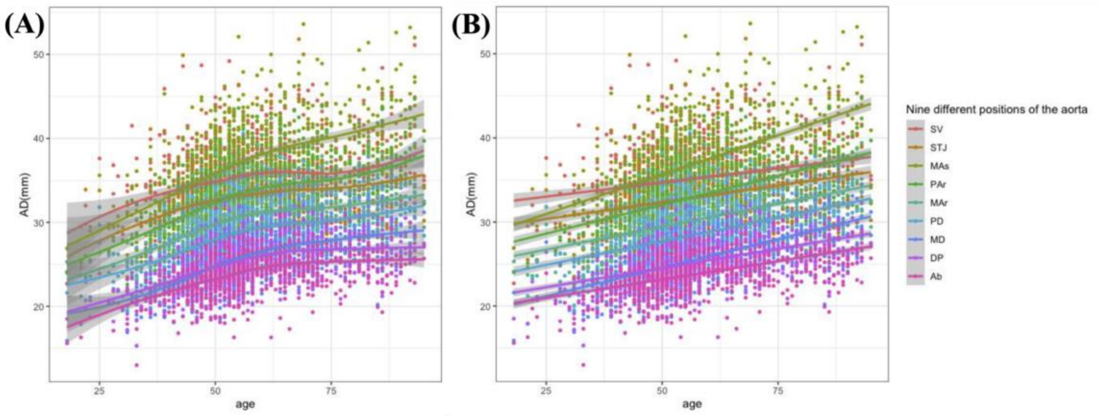

(A)The scattergram fitted by LOESS, with age as the independent variable, and ADs as the dependent variable

(B) The scattergram fitted by linear regression, with age as the independent variable, and ADs s the dependent variable

Abbreviations are the same as Fig2

Supplementary Figure 4 The three-dimensional scatter plot of the interaction association among the three variables of age, M.CAVI/M.CAVI$_0$, and the AD at the level of MD

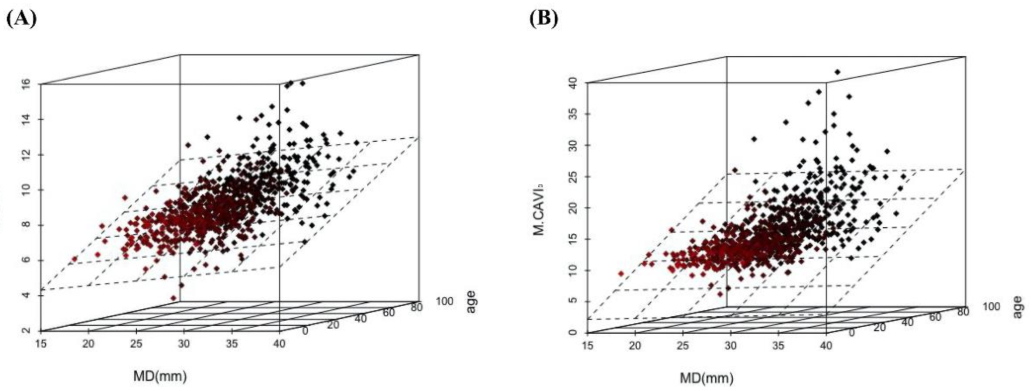

(A) A three-dimensional scatter plot with AD at MD as the x-axis, age as the y-axis, and M.CAVI as the z-axis

The plane is fitted by regression equation (M.CAVI~MD + age)

(B) A three-dimensional scatter plot with AD at MD as the x-axis, age as the y-axis, and M.CAVI$_0$ as the z-axis

The plane is fitted by regression equation (M.CAVI$_0$~MD + age)

As the fitted plane rises from the lower left to the upper right. The darker the scatter, the larger the value.

Abbreviations are the same as Fig2
